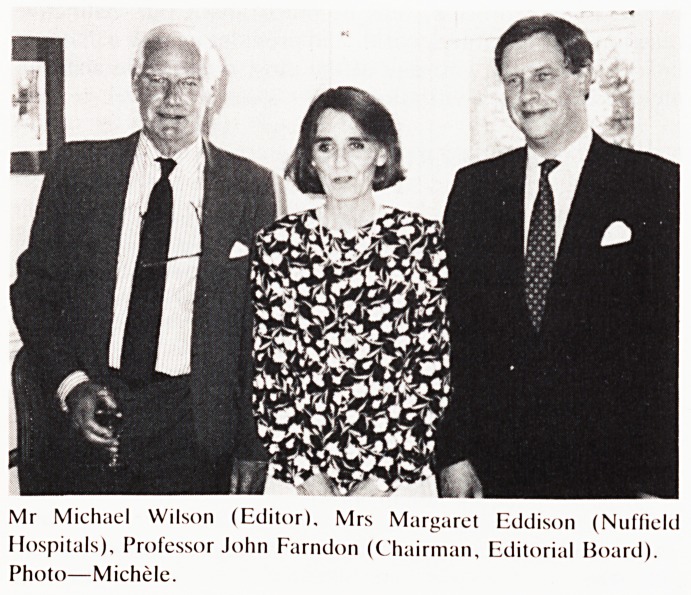# Launch of the 'New Journal'

**Published:** 1990-06

**Authors:** 


### Launch of the New Journal

A party to celebrate the birth of the new *West of England Medical Journal* and to launch it on its career was generously given by our sponsors, Nuffield Hospitals at the Chesterfield Hospital, Bristol on May 31st 1990.

The event was well attended and a toast to the success of the new journal in champagne began our career in style.

The Chairman of the Editorial Board, Professor John Farndon said that the *West of England Medical Journal* was built on the centenarian Bristol Medico-Chirurgical Journal which at its inception in 1883 had stated its aim to be "A journal of the Medical Sciences for the West of England and South Wales". It had, however, remained the journal of the Bristol Medico-Chirurgical Society and until recently had only a small distribution. Now the Bristol Medico-Chirurgical Society had formed an association with other West of England Medical groups and the new journal was the product of this association and would be distributed to all its members. They in turn would provide it with material and it would become a genuine regional medical journal. It is doubtful whether this association could have been achieved if the considerable cost of producing this journal had not been underwritten by Nuffield Hospitals and he wished to express gratitude to them for their generosity and he hoped that the quality of the journal would give them the satisfaction of feeling that their money was being well spent.

Mr Michael Wilson (Editor), Mrs Margaret Eddison (Nuffield Hospitals), Professor John Farndon (Chairman, Editorial Board). Photo—Michèle.

On behalf of Nuffield Hospitals, Mrs Margaret Eddison said that Nuffield Hospitals is an independent charity whose aims were not only to provide a network of private hospitals throughout the country but also to contribute to medical knowledge and education, they believed that sponsoring this journal would foster these aims, they were very glad that they had entered into this partnership and had confidence that the aims would be achieved.

Mr Michael Wilson, editor of the new journal and formerly editor of its predecessor the Bristol Medico-Journal stated the aims of the journal—to produce a broadly based general medical journal in which there would be something for everybody, to provide a forum for original work especially for West of England sources, publish clinical articles, report medical news from the region, give West of England doctors an opportunity to express their views on events, publish abstracts of papers given at meetings of the various member groups, publish papers on medical history, on doctor's hobbies and their own special brand of humour and views on life in general. In short any contribution from a doctor on whatever subject would be considered if it was good enough. He used the opening number as a specimen and hoped that many of these aspects had been covered. He also expressed gratitude to Nuffield Hospitals for their crucial assistance and for their confidence and wished to thank Mrs Eddison especially because she had personally done a very great deal to bring about this partnership.

## Figures and Tables

**Figure f1:**